# Chemokine receptor CX3CR1 contributes to macrophage survival in tumor metastasis

**DOI:** 10.1186/1476-4598-12-141

**Published:** 2013-11-18

**Authors:** Jiao Zheng, Min Yang, Jianghua Shao, Yanju Miao, Jiahuai Han, Jie Du

**Affiliations:** 1 Beijing Anzhen Hospital, Capital Medical University, Beijing, China; 2 The Key Laboratory of Remodeling-Related Cardiovascular Diseases, Capital Medical University, Ministry of Education, Beijing Institute of Heart Lung and Blood Vessel Diseases, Beijing 100029, China; 3 The Second Affiliated Hospital to Nanchang University, Jiangxi 330006, China; 4 School of Life Sciences, Xiamen University, Fujian 361005, China

**Keywords:** CX3CR1, Tumor metastasis, Macrophage, Apoptosis, Angiogenesis

## Abstract

**Background:**

Macrophages, the key component of the tumor microenvironment, are differentiated mononuclear phagocyte lineage cells that are characterized by specific phenotypic characteristics that have been implicated in tumor growth, angiogenesis, and invasion. CX3CR1, the chemoattractant cytokine CX3CL1 receptor, plays an important role in modulating inflammatory responses, including monocyte homeostasis and macrophage phenotype and function. However, the role of CX3CR1 in the regulation of the tumor inflammatory microenvironment is not fully understood.

**Methods:**

Using *in vivo* hepatic metastasis model, human colon carcinoma specimens, immunohistochemical staining, TUNEL staining, flow cytometry analysis, Western blotting assay and co-culture in three-dimensional peptide gel, we determined the effects of CX3CR1 on angiogenic macrophage survival and tumor metastasis.

**Results:**

In this study, we found that CX3CR1 was expressed in human colon carcinomas in a histologic grade- and stage-dependent manner, and CX3CR1 upregulation in TAMs was correlated with poor prognosis. Furthermore, we showed that in a microenvironment lacking CX3CR1, the liver metastasis of colon cancer cells was significantly inhibited. The underlying mechanism is associated with decrease accumulation of angiogenic macrophages that can be partly attributed to increased apoptosis in the tumor microenvironment, thus leading to impaired tumor angiogenesis in the liver and suppressed tumor metastasis.

**Conclusions:**

Our results suggest a role of CX3CR1 in angiogenic macrophage survival in the tumor microenvironment contributing to tumor metastasis.

## Background

Tumors exhibit a complex cellular ecology that establishes their malignant potential. In addition to genetic complexity, it has become increasingly apparent that the tumor inflammatory microenvironment plays an active role in promoting all stages of tumor progression [1-3]. Tumor ecosystems contain innate immune cells, the most abundant of which are macrophages. Although the original hypotheses proposed that macrophages are involved in antitumor immunity, there is substantial clinical and experimental evidence suggesting that tumor-associated macrophages (TAMs) enhance tumor progression to malignancy in the majority of cases [4,5]. The tumor-promoting functions of macrophages include supporting tumor-associated angiogenesis, promoting tumor cell invasion and migration, and suppressing antitumor immune responses [6]. Accumulating evidence suggests that tumor initiation, progression, and metastasis are affected by dynamic changes in the phenotypes of macrophages, and that defined subpopulations of macrophages are responsible for these tumor-promoting activities. Inflammation and angiogenesis are the hallmarks of cancer. Infiltration of TAMs is associated with a poor prognosis [7], and studies have shown that not only their numbers but also their phenotype regulate tumor progression. However, the regulatory mechanisms of the phenotype and function of TAMs remain elusive.

In this study, we explored whether CX3CR1, a chemoattractant cytokines receptor, regulates the TAMs subtypes in the tumor microenvironment, for several reasons. First, CX3CR1 is primarily expressed on circulating monocytes, tissue macrophages, and tissue dendritic cell populations but is also expressed on T cell and natural killer cell subsets [8]. Second, it is generally accepted that the surface molecule CX3CR1 can be used to identify monocyte subsets owing to its differential expression [9,10]. Third, CX3CR1 and its ligand help control the migration and recruitment of immune effector cells in numerous inflammatory diseases and may play a role in cancer progression, immune evasion, and metastasis [8,11,12]. Fourth, increasing evidence indicates that CX3CR1 is required for monocyte homeostasis and differentiation and regulates the fate of monocyte-derived cells in other inflammatory diseases such as cardiovascular disease and liver fibrosis [13-15]. However, precisely how CX3CR1 regulates TAMs subtypes in the tumor microenvironment remains unknown.

Our study showed that enhanced CX3CR1 expression was correlated with the poor prognosis in human colon carcinoma. In mice lacking CX3CR1, the liver metastasis of colon cancer cells was significantly inhibited. Mechanistically, CX3CR1 signaling was critical for survival of angiogenic macrophages and promoting tumor angiogenesis. This work highlights the function of CX3CR1 in the tumor inflammatory response and identifies CX3CR1 signaling as the important player in the complex interactions between tumor cells and the tumor microenvironment.

## Results

### Expression of CX3CR1 in TAMs is increased along with colon carcinoma development

**Figure 1:**
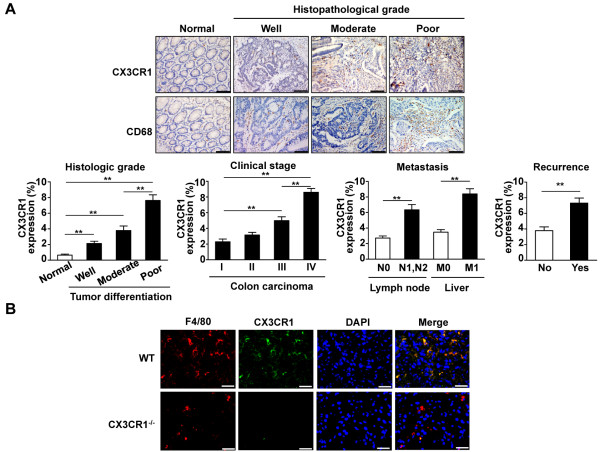
**Expression of CX3CR1 in tumor tissues. (A)** Immunohistochemical analysis of CX3CR1 or macrophage marker CD68 expression in normal colon tissues and human colon carcinoma. Histopathological differentiation grade indicates well-, moderately- and poorly-differentiated. T indicates tumor invasion depth. N indicates lymph node metastasis. M indicates distant metastasis. (×200 magnification and Scale bars = 100 μm). Quantitative analysis of CX3CR1 expression in tumor sections at different histologic grade (well, moderate and poor), different tumor stages (I-IV) and with or without recurrence 3 years after operation (n = 5-10 samples per group, with 10 fields per samples). **, *P*<0.01. **(B)** Double-color immunofluorescence analyses of macrophage and CX3CR1 expression in tumors. Tumor tissues of metastasized foci in the liver were obtained from WT and CX3CR1^−/−^ mice at 14 days after intrasplenic SL4 cells injection. The sections were immunostained with anti-F4/80 and anti-CX3CR1 antibodies. Three independent experiments were performed. (×400 magnification and Scale bars = 50 μm).

Because the treatment and prognosis of colorectal cancer patients depends on the tumor grade (degree of primary tumor differentiation) and tumor-node-metastasis stage (how widespread the cancer is at the time of diagnosis), we first measured the CX3CR1 expression levels in normal colon tissues and human colon cancer tissue. As shown in Figure 1A, CX3CR1 was rarely detected in normal tissues, whereas CX3CR1 was observed in the inflammatory-like cells located in carcinoma tissue. CX3CR1 expression was significantly elevated in poorly differentiated colon carcinoma, which was much higher than that of moderately differentiated or well-differentiated colon carcinoma (Figure 1A). The clinical colon carcinoma stages were characterized by extensive invasion of the primary tumor into the submucosa, muscularis propria, or through the wall of the colon, and by invasion to nearby lymph nodes at stage III and to distant metastases in stage IV tumors. CX3CR1 was expressed in a high percentage of stage III and IV tumors (Figure 1A). Moreover, analysis of CX3CR1 expression in primary tumors and formation of metastases revealed that patients who had advanced clinical stage disease, metastasis, or recurrent lesion within 3 years showed increased CX3CR1 expression in colon cancer tissue (Figure 1A). Thus, CX3CR1 expression is associated with the aggressiveness of human colon cancer.

In addition, the advanced colon carcinoma tissues were markedly and extensively infiltrated by CD68-positive macrophages, particularly along the tumor cell-invasive front; however, the number of macrophages in normal colon tissues was low (Figure 1A). CX3CR1-positive cells within the area colocalized with CD68-positive macrophages, indicating a high concordance of CD68 and CX3CR1 expression in human primary colon carcinoma during malignant progression.

To further analyze the expression of CX3CR1 in the tumor stroma, we utilized a murine model in which mouse colon cancer cells (SL4) were injected into the spleen and tumors developed in the liver. Previous studies have shown that SL4 cells form tumors in the liver of 100% of syngeneic mice when injected intrasplenically. This model is not a pathological metastatic tumor model; however, SL4 cells are a useful tool to study how injected colon carcinoma cells seed and develop into a new microenvironment in the liver [16]. Liver tumor sections were immunostained with the macrophage marker F4/80 and an anti-CX3CR1 antibody. Immunofluorescence staining showed that CX3CR1 was abundantly expressed in macrophages in hepatic metastatic tumors of WT mice; as expected, there was no CX3CR1 expression in CX3CR1^−/−^ control mice (Figure 1B). These results indicate that expression of CX3CR1 in TAMs is increased during malignant progression, which might have predictive value in determining tumor outcome.

### CX3CR1 deficiency impairs the hepatic metastatic ability of colon cancer cells

**Figure 2:**
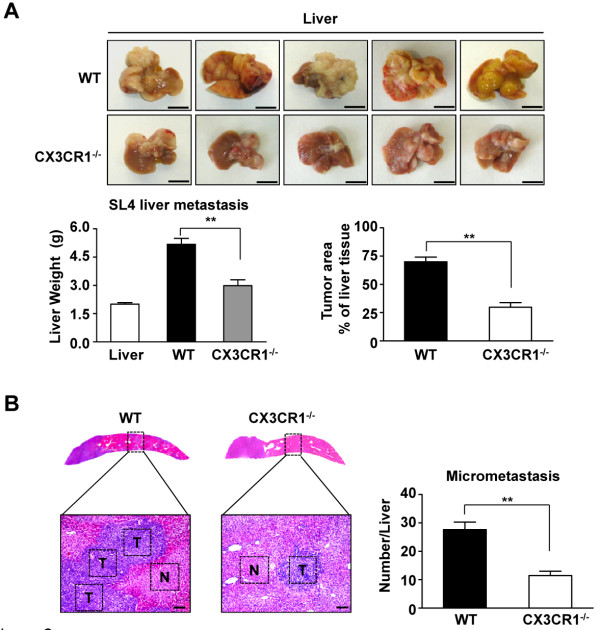
**CX3CR1 deficiency inhibits metastatic ability of colon cancer cells. (A)** Gross examination of development of liver metastasized tumor of colon cancer 14 days after intrasplenic injection of SL4 cells in WT and CX3CR1^−/−^ mice. SL4 cells (1 × 10^6^) were injected into the spleen of WT and CX3CR1^−/−^ mice. Mice were sacrificed at 14 days after tumor injection to determine the incidence of liver metastasis, the number of metastasized foci in the liver, tumor weight and total tumor area represented as% of liver tissue. Data are mean ± SEM for n = 10 mice **, *P*<0.01. **(B)** The analysis of micrometastasis was performed on paraffin-embedded sections with HE staining in metastasized foci after intrasplenic injection of SL4 cells in WT and CX3CR1^−/−^ mice. T, tumor tissue; N, normal liver tissue. (×100 magnification and Scale bars = 100 μm). Data are mean ± SEM for n = 10 mice with 10 fields per animal. **, *P*<0.01.

To investigate the role of CX3CR1 in tumor development of colon carcinoma cells in the liver, we injected CX3CR1^−/−^ mice and WT controls with SL4 cells intrasplenically. The SL4 cells generated tumors within the spleen (primary) and liver (metastasis). Multiple hepatic tumor nodules, detectable by gross inspection, were evident by 2 weeks. Livers were isolated and the incidence of hepatic metastasis was evaluated. Livers from CX3CR1^−/−^ mice showed significantly smaller tumor foci, fewer metastatic tumors, and decreased tumor-occupied area compared with those from tumor-bearing WT mice (Figure 2A).

To further evaluate the anti-metastasis effect in CX3CR1 deficiency, micrometastasis to livers were quantified in H&E-stained sections. WT mice exhibited massive and developed metastatic nodules in their livers, whereas CX3CR1^−/−^ mice had several small metastasis nodules (Figure 2B). Histologic analysis showed that the number of hepatic micrometastasis was significantly decreased in CX3CR1^−/−^ mice compared with the number in WT mice. These results indicate that CX3CR1 plays a critical role in promoting metastasis.

### CX3CR1 deficiency reduces macrophage accumulation in metastatic tumors

**Figure 3:**
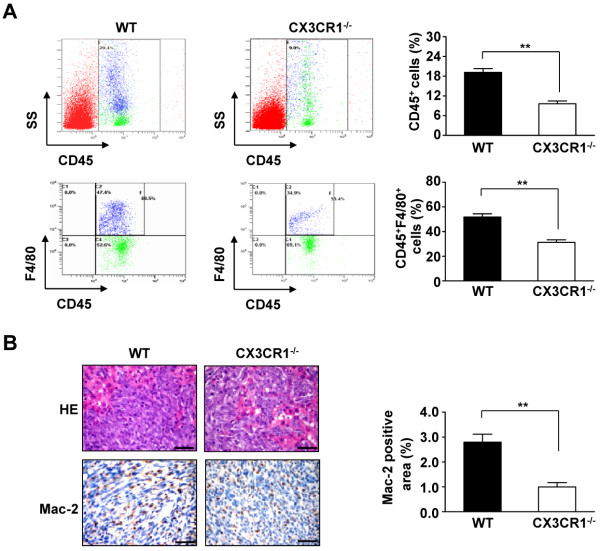
**CX3CR1 deficiency reduces macrophages accumulation in metastasized tumor. (A)** Leukocytes were gated with CD45 fluorescence vs side angle scatter (SS). Scatter plots are gated on CD45^+^ population cells. Macrophages (CD45^+^F4/80^+^ cells) were detected by flow from metastasized foci in the liver after intrasplenic injection of SL4 cells in WT and CX3CR1^−/−^ mice. Data represent the means ± SEM for n = 8 mice. **, *P*<0.01. **(B)** HE staining and immunohistochemical analysis of macrophage infiltration in metastasized foci after intrasplenic injection of SL4 cells in WT and CX3CR1^−/−^ mice. Macrophage infiltration detected by anti-Mac-2 immunostaining. (×400 magnification and Scale bars = 50 μm). Quantitative analysis of Mac-2 positive cells in metastasized foci sections. Data are mean ± SEM for n = 8 mice with 10 fields per animal. **, *P*<0.01.

The relative proportions of leukocytes that infiltrated into hepatic metastatic tumors were analyzed and compared between CX3CR1^−/−^ and WT mice. As shown in Figure 3A, the proportion of CD45-positive leukocytes that infiltrated into metastatic tumors was significantly decreased in CX3CR1^−/−^ mice compared to that in WT mice (9.61 ± 0.87% versus 19.16 ± 1.12% for CD45, *P*<0.01). In particular, the proportion of infiltrating CD45^+^F4/80^+^ cells in metastatic tumors was significantly lower in CX3CR1^−/−^ mice than that in WT mice (31.22 ± 2.10% versus 51.79 ± 2.66% for CD45^+^F4/80^+^ cells, *P*<0.01; Figure 3A). Furthermore, immunohistochemical staining of metastatic foci further demonstrated that the expression of Mac-2 (a macrophage marker) was markedly decreased in CX3CR1^−/−^ mice relative to that in WT mice (Figure 3B). Thus, CX3CR1 plays an important role in promoting macrophage infiltration in metastatic tumors.

### CX3CR1 deficiency enhances macrophage apoptosis in metastatic tumors

**Figure 4:**
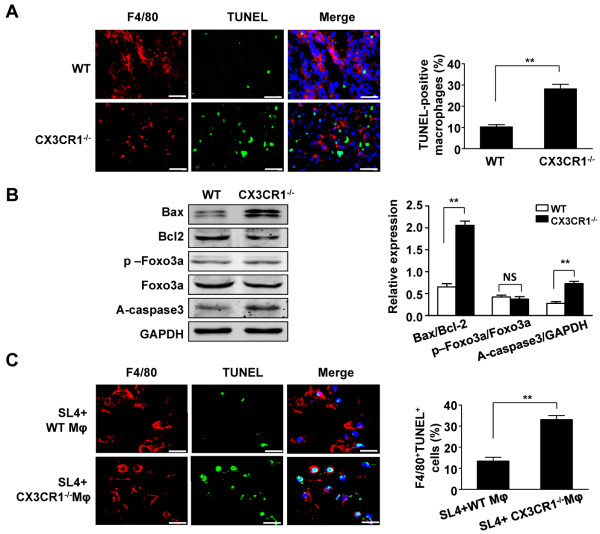
**CX3CR1 deficiency increases macrophage apoptosis in metastasized tumor. (A)** The TUNEL assay and immunofluorescence analysis of macrophage apoptosis from metastasized foci after intrasplenic injection of SL4 cells in WT and CX3CR1^−/−^ mice. Representative micrographs show F4/80-positive (red), TUNEL-positive signal (green), nuclei (blue) in metastatic foci. The apoptotic index of macrophages was assessed as the percentage of apoptotic TUNEL^+^F4/80^+^ cells to the total number of F4/80^+^ cells. Scale bars =40 μm. Data are mean ± SEM for n = 8 mice with 10 fields per animal. **, *P*<0.01. **(B)** Western blot analysis of the protein levels of Bax, Bcl-2, phospho-FOXO3a, FOXO3a and activate caspase3 from metastasized foci in the liver after intrasplenic injection of SL4 cells in WT and CX3CR1^−/−^ mice. GAPDH was used as a loading control. Quantitative analysis of Bax/ Bcl-2 ratio, phospho-FOXO3a/FOXO3a ratio and activate caspase3 expression in metastasized foci from WT and CX3CR1^−/−^ mice. Data are mean ± SEM for n = 8 mice. **, *P*<0.01. NS, not significant. **(C)** WT or CX3CR1^−/−^ macrophages were co-cultured with SL4 cells in peptide gels and then underwent TUNEL assay and immunofluorescence analysis of macrophage apoptosis by staining with F4/80 antibody. The nucleus was stained with DAPI. Representative micrographs show F4/80-positive (red), TUNEL-positive signal (green), nuclei (blue) in coculture. The apoptotic index of macrophages was assessed as the percentage of apoptotic TUNEL^+^F4/80^+^ cells to the total number of F4/80^+^ cells from 10 randomly selected regions. Scale bars = 25 μm. Data are mean ± SEM of 3 independent experiments. **, *P*<0.01. Mφ indicates macrophages.

To investigate the mechanism by which CX3CR1 promotes macrophage infiltration into metastatic foci, we analyzed macrophage apoptosis in metastatic tumors. Both the number of TUNEL- and F4/80-positive (macrophage marker) cells was significantly increased in metastatic tumors in CX3CR1^−/−^ mice compared with those in WT mice (Figure 4A). Moreover, we analyzed the role of CX3CR1 on apoptotic pathways. As shown in Figure 4B, CX3CR1 knockout significantly increased the Bax/Bcl-2 ratio in metastatic tumors compared with that in WT mice. Similarly, caspase 3 activation was markedly unregulated in the metastatic tumors of CX3CR1^−/−^ mice versus WT mice (Figure 4B). However, no alteration of FOXO3a phosphorylation was detected in metastatic foci in either CX3CR1^−/−^ or WT mice (Figure 4B).

To understand the role of CX3CR1 in macrophage survival during hepatic metastasis of colon cancer, we cocultured macrophages derived from CX3CR1^−/−^ or WT mice with murine SL4 colon cancer cells in a 3D peptide gel. A peptide gel was chosen because it more accurately resembles the extracellular matrix milieu of carcinoma compared to Matrigel, which models the laminin-rich basement membrane-type extracellular matrix [17]. As shown in Figure 4C, the number of both TUNEL- and F4/80-positive (macrophage marker) cells was significantly increased in CX3CR1^−/−^ macrophages cocultured with SL4 cells, but not WT macrophages. Therefore, CX3CR1 is responsible for promoting macrophage survival within the tumor microenvironment, which is typically associated with TAMs that contribute to tumor development.

### Expression of CX3CR1 in macrophages promotes angiogenesis in metastatic tumors

**Figure 5:**
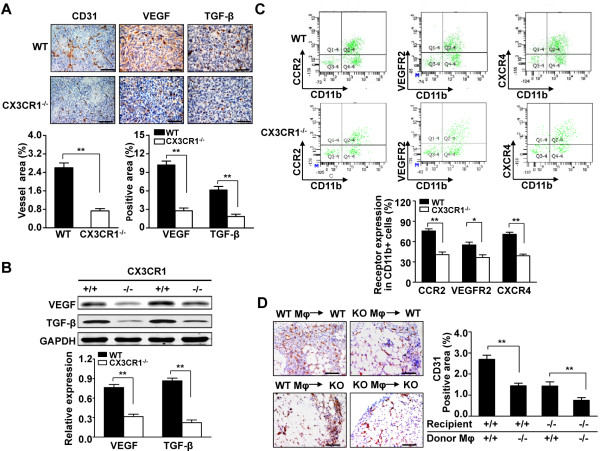
**CX3CR1 deficiency blocks angiogenesis through inhibiting angiogenic macrophage in tumor. (A)** The area of vessels and the expression of pro-angiogenesis cytokines were evaluated by immunohistochemical analysis with anti-CD31, anti-VEGF or anti-TGF-β antibodies. (×200 magnification and Scale bars = 100 μm). Quantitative analysis of CD31, VEGF and TGF-β expression in metastasized foci sections. Data are mean ± SEM for n = 8 mice with 10 fields per animal. **, *P*<0.01. **(B)** Western blot analysis of the protein levels of VEGF and TGF-β in metastasized foci. GAPDH was used as a loading control. Quantitative analysis of VEGF and TGF-β expression in metastasized foci from WT and CX3CR1^−/−^ mice. Data are mean ± SEM for n = 8 mice. **, *P*<0.01. **(C)** Scatter plots gated on CD45^+^ cells, angiogenic markers CCR2, VEGFR2 and CXCR4 in CD11b^+^ cells were determined from metastasized foci in the liver after intrasplenic injection of SL4 cells in WT and CX3CR1^−/−^ mice. Data are mean ± SEM for n = 8 mice. *, *P*<0.05; **, *P*<0.01. **(D)** Matrigel plugs containing either WT or CX3CR1^−/−^ macrophages were subcutaneously implanted in WT or CX3CR1^−/−^ mice for 7 days. Evaluation of blood vessels with anti-CD31 antibody. (×200 magnification and Scale bars = 100 μm). Data are mean ± SEM of 3 independent experiments with 10 fields per sample. **, *P*<0.01. Mφ indicates macrophages.

We investigated whether the decreased metastatic ability of tumor cells in CX3CR1^−/−^ mice could be caused by inhibition of angiogenesis. Thus, we stained slices of tumors of comparable size, grown either in WT or CX3CR1^−/−^ mice, with an anti-CD31 antibody (a marker of endothelial cells). As shown in Figure 5A, the total vessel area and size were significantly smaller in metastatic foci grown in the livers of CX3CR1^−/−^ mice than those in WT mice. Immunohistochemistry and western blot analysis showed that the levels of the proangiogenic cytokines VEGF and TGF-β were significantly decreased in metastatic liver tumors in CX3CR1^−/−^ mice compared with those in WT mice (Figure 5A and B). To further explore the relationship between CX3CR1 expression in macrophages and tumor angiogenesis, we assayed the proangiogenic function of macrophages from metastatic tumors. Flow cytometric analysis revealed that macrophages from metastatic tumors in CX3CR1^−/−^ mice expressed significantly less CCR2, VEGFR2, and CXCR4 (proangiogenic markers) compared to WT mice (40.72 ± 3.89% versus 75.78 ± 3.08% for CCR2, *P*<0.01; 36.58 ± 3.97% versus 55.08 ± 4.13% for VEGFR2, *P*<0.05; 39.04 ± 2.46% versus 70.78 ± 3.07% for CXCR4, *P*<0.01; Figure 5C).

To further confirm the *in vivo* proangiogenic role of CX3CR1 expression in macrophages, Matrigel plugs containing WT or CX3CR1^−/−^ macrophages were subcutaneously implanted in WT or CX3CR1^−/−^ mice. After 7 days, the mice were sacrificed and the plugs were microscopically analyzed by staining the blood vessels with an anti-CD31 antibody. As shown in Figure 5D, plugs containing WT macrophages in WT mice were well vascularized with well-formed and branched vessels, whereas plugs containing CX3CR1^−/−^ macrophages in WT mice displayed poorly organized vessels. In addition, plugs containing WT macrophages in CX3CR1^−/−^ mice showed well-formed vessels compared to those containing CX3CR1^−/−^ macrophages in CX3CR1^−/−^ mice. Taken together, these results suggested that CX3CR1 was necessary for TAMs-induced angiogenic responses during tumor development.

## Discussion

The chemokine receptor CX3CR1 plays an important role in the development of numerous chronic inflammatory diseases by modulating inflammatory responses, particularly macrophage phenotype and function. As cancer is a chronic inflammatory disease, it is recognized that the inflammatory microenvironment plays a critical role in tumor progression. Recent studies have demonstrated that the monocyte/macrophage chemokine receptor CX3CR1 is essential for nascent microvessel formation, structural integrity and maturation in Matrigel and experimental plaque neovascularization models [18]. Although CX3CR1 plays a positive role in neovascularization, the mechanism by which CX3CR1 regulating macrophage function contributing to tumor development remains unclear. In the present study, especially we demonstrated the role of CX3CR1 in regulating tumor inflammatory microenvironment. The present work elucidated CX3CR1 mediates survival of macrophage, promoting angiogenesis leading to tumor metastasis.

Tumor development is a complex event that involves not only tumor cells but also the surrounding stroma. The tumor microenvironment and neoplastic cells act in concert to promote the growth and progression of the tumor mass [4]. In the stroma of several tumor types, a critical role has been demonstrated for TAMs, which represent the major inflammatory component [5,19]. Experimental models have demonstrated that the lack of macrophage recruitment to the tumor site results in decreased tumorigenic ability [20,21], and clinical evidence has shown a correlation between high TAMs content inside of tumors and a poor prognosis [19]. Along with these previous results, our study showed that macrophage infiltration was negatively associated with human colon carcinoma prognosis (Figure 1A).

TAMs are differentiated mononuclear phagocytic lineage cells that are characterized by specific phenotypic characteristics and the expression of particular markers, and have been implicated in tumor growth, invasion, and angiogenesis in the tumor microenvironment [6,22-25]. CX3CR1 has been used to identify macrophage subsets owing to its differential expression; this surface molecule plays an important role in the initiation and progression of inflammation [9-12,26] and is upregulated in inflammatory diseases [8,27-31]. However, the role of CX3CR1 in the development of the tumor microenvironment remains unclear. In this study, we observed that colon carcinoma patients at more advanced clinical stages, those with lymph node or liver involvements, and those who had recurrence within 3 years displayed markedly higher CX3CR1 expression levels (Figure 1A). The association between the increased expression of CX3CR1 and clinical stage, metastasis, and recurrence suggests its potential utility as an independent or supplementary biomarker in the prediction of tumor prognosis. Furthermore, we found that development of metastatic liver tumors was substantially inhibited in CX3CR1^−/−^ mice as compared with WT littermates, suggesting the critical role of CX3CR1 expression in TAMs-mediated tumor development.

The chemokine receptor CX3CR1 is the only known corresponding CX3CL1 receptor. CX3CR1 is primarily expressed on circulating monocytes, tissue macrophages, and tissue dendritic cell populations but is also expressed on T cell and natural killer cell subsets [8]. In this study, we showed that the absence of CX3CR1 reduced the proportion of macrophages that infiltrated into the metastatic foci (Figure 2). CX3CR1 is required for monocyte survival and differentiation in atherosclerosis and liver fibrosis [13-15]. We observed that CX3CR1 deficiency significantly promoted macrophage apoptosis in the tumor microenvironment both *in vivo* and *in vitro* (Figure 4), which indicates that CX3CR1 deficiency inhibits macrophage infiltration in metastatic tumors through induction of macrophage apoptosis. Studies have demonstrated that apoptosis is regulated by a complex network of signaling pathways that control the expression and degradation of key molecules, including Bcl-2 family proteins and caspases [32]. As expected, CX3CR1 promoted the survival of macrophages in metastatic tumors through suppression of the proapoptotic pathway (Figure 4B).

In most tumors, there is dramatic enhancement of vascular density from the benign-to-malignant transition, a process referred to as the angiogenic switch. These data strongly argue for a role of the angiogenic switch in regulating malignant transition and for macrophages as important players in vascular remodeling as tumors progress to late carcinoma stages [20,33-35]. This effect seems to be the most likely cause of reduced tumor growth and metastasis after the macrophage depletion observed in these transplants models, as tumor progression is closely dependent on rapid angiogenesis. We therefore wondered if CX3CR1 could play a role in tumor angiogenesis. Our results showed that CX3CR1 deficiency impaired tumor angiogenesis in metastatic foci (Figure 5A and B). Furthermore, our data demonstrated that CX3CR1 deficiency suppressed macrophage expression receptors CCR2, VEGFR2, and CXCR4 (cell surface markers of angiogenic macrophages) [6] in hepatic metastatic tumors (Figure 5C). We further confirmed that CX3CR1 expression in macrophages was required for angiogenesis *in vivo*, as its deficiency resulted in the presence of smaller vessel density and vessel area (Figure 5D). Taken together, these data suggested that CX3CR1 contributed to macrophages survival and promoting angiogenesis in the tumor microenvironment.

## Conclusions

In summary, we identified the role for CX3CR1 in macrophages that can promote tumor metastasis and is associated with poor cancer prognosis by contributing to angiogenic macrophage survival. As numerous attempts are ongoing to target the tumor stroma, we propose CX3CR1 as a potential molecular target for antiangiogenic and antimetastatic cancer therapy.

## Methods

### Antibodies and reagents

The antibodies for CX3CR1, CD68, CD31, VEGF, TGF-β, Mac-2, GAPDH, and IgG were from Santa Cruz Biotechnology (Santa Cruz, CA, USA); the antibodies for phospho-FOXO3a, FOXO3a, Bax and Bcl-2 were from Cell Signaling Technology (Beverly, MA, USA); the antibodies for F4/80 and A-Caspase3 were from Abcam (Cambridge, MA, USA); and ChemMate TM EnVision System/DAB Detection Kits were from Dako (Glostrup, Denmark). Antibodies for PerCP/Cy5.5-conjugated CD45.2, phycoerythrin (PE)-conjugated F4/80, APC-conjugated CD11b and isotype control were from Biolegend (San Diego, CA, USA). Antibodies for PE-conjugated CCR2, PE-conjugated VEGFR2, PE-conjugated CXCR4 and isotype control were from R&D Systems (Minneapolis, MN, USA). DeadEnd™ fluorometric TUNEL system was from Promega (Madison, WI, USA).

### Animals

CX3CR1 knockout (CX3CR1^−/−^) mice and wild-type (WT) littermates were used for all experiments. CX3CR1 knockout (CX3CR1^−/−^) mice were harbored a target replacement of the CX3CR1 gene by a GFP gene [36]. CX3CR1^−/−^ mice were obtained from the Jackson Laboratory and maintained in a Specific Pathogen-Free atmosphere at the Beijing Anzhen Hospital affiliated to the Capital Medical University, China. The investigations conformed to the US National Institutes of Health Guide for the Care and Use of Laboratory Animals (publication no. 85–23, 1996) and were approved by the Animal Care and Use Committee of Capital Medical University.

### Human colon carcinoma specimens

The specimens from 30 cases of human colon carcinoma tissue/adjacent normal colon tissues and the clinicopathologic data were obtained from the Second Affiliated Hospital to Nanchang University gastrointestinal tumor bank. The specimens were isolated at the time of surgery, formalin-fixed and paraffin-embedded, and stained with hematoxylin and eosin, then examined by 2 experienced pathologists. The clinicopathologic stage was determined according to the TNM classification system of the International Union against Cancer. Human specimens use for research had been approved by the Second Affiliated Hospital to Nanchang University Research Ethics Committee.

### Tumor model

A highly metastatic murine colon adenocarcinoma cell line, colon SL4, was used for the *in vivo* experiments [16]. SL4 cells were derived from C57BL/6 mice on the same background as the CX3CR1 deficient mice and WT control mice. SL4 cells were maintained in 1:1 mixture of Dulbecco’s modified Eagle’s medium and Ham’s F-12 medium (DMEM/F12) containing 10% heat-inactivated fetal calf serum (FCS) in a humidified atmosphere of 95% air and 5% CO_2_ at 37°C. For *in vivo* model, after anaesthetizing mice, a transverse incision in the left flank was made, exposing the spleen, then 1.0 × 10^6^ SL4 tumor cells in 100 μl DMEM/F12 medium were intrasplenically injected with use of a 26-gauge needle. 14 days after inoculation, mice were sacrificed, and the tissues were processed as described below. After the initial dissection, the left ventricle was flushed by puncture with 40 ml normal saline to remove blood samples. The spleen and liver were removed, wet spleen and liver weights were measured, and the incidence of liver tumor development was examined.

### Histology and immunohistochemistry

Livers in mice were fixed for 24 hrs with 10% buffered formalin before embedding in paraffin. Serial sections of 5 μm thick were obtained for histologic analysis. Hematoxylin&eosin (HE) staining involved standard procedures.

For immunohistochemistry, sections were incubated with the primary antibody for CX3CR1(1:200), CD68(1:200), CD31(1:200), VEGF(1:200), TGF-β(1:200), or Mac-2(1:200), then incubated with the Dako ChemMate™ EnVision System (Dako, Glostrup, Denmark) for 30 mins. Staining was visualized with use of diaminobenzidine and counterstaining with hematoxylin. Negative controls were omission of the primary antibody, non-immune IgG or secondary antibody only; in all cases, negative controls showed insignificant staining. Images were captured with use of a Nikon Labophot 2 microscope equipped with a Sony CCD-Iris/RGB color video camera attached to a computerized imaging system and analyzed by use of ImagePro Plus 3.0 (ECLIPSE80i/90i; Nikon, Japan) with blinding to treatment. The expression of CX3CR1, CD68, CD31, VEGF, TGF-β, or Mac-2 was calculated as proportion of positive area to total tissue area for all measurements of the section.

For double immunofluorescence, tumors were excised and fixed in 4% paraformaldehyde for 2 hrs, then dehydrated with 30% sucrose/PBS and frozen in compound. Frozen tissue sections, 7 μm, were permeabilized and blocked with 0.1% Triton X-100, 0.2% bovine serum albumin, and 5% normal donkey serum in PBS, then incubated with the primary antibodies F4/80 (1:100) and CX3CR1 (1:100)overnight at 4°C, then FITC or TRITC-conjugated secondary antibody (Jackson Immuno Research Laboratories, West Grove, PA, USA) at room temperature for 1 hr in the dark, and coverslipped with DAPI-containing mounting medium.

### TUNEL staining

Identification of apoptotic cells was performed using TUNEL system Kit (Promega). Briefly, frozen slides were permeabilized using 0.1% solution of Triton X-100 in sodium citrate. Consequently, slides were incubated with TUNEL reaction mixture for 1 hr at 37°C. To allow detection of apoptotic macrophages, frozen sections were incubated with the primary antibodies for F4/80 (1:100) at 4°C overnight and then with TRITC-conjugated secondary antibody (Jackson ImmunoResearch Laboratories) at room temperature for 1 hr. Nuclei were stained with DAPI (Invitrogen). The apoptotic index of macrophages was assessed as the percentage of apoptotic TUNEL^+^ F4/80^+^ cells to the total number of F4/80^+^ cells.

### Flow cytometry

For extracellular staining of immune markers, single-cell suspensions were prepared by mechanical dispersion and enzymatic digestion of tumor tissues as described [17]. Briefly, tumor tissues were cut into multiple small cubes and digested in an enzyme mixture for 45 mins at 37°C. The cell suspension was centrifuged and preincubated with Fc-γ block antibody (anti-mouse CD16/32; Pharmingen, San Diego, CA, USA) to prevent nonspecific binding. Cell staining involved different combinations of fluorochrome-coupled antibodies to CD45, F4/80, CD11b, CCR2, VEGFR2 or CXCR4 for 30 mins at room temperature in the dark. Fluorescence data were collected by use of an EPICS XL flow cytometer (Beckman Coulter) and analyzed by use of Cellquest (Beckman). Fluorescence minus one (FMO) controls were included to determine the level of nonspecific staining and autofluorescence associated with subsets of cells in each fluorescence channel.

### Western blotting

Protein extracts were were diluted with loading buffer and separated by electrophoresis on 10% SDS-polyacrylamide gels before transfer to nitrocellulose membranes. The membranes were blocked in Odyssey blocking buffer (LI-COR Bioscience, Lincoln, NE) at room temperature for 1 hr, then incubated at 4°C overnight with primary antibody: Bax (1:500), Bcl2 (1:500), p-Foxo3a (1:800), Foxo3a (1:800), A-caspase3 (1:800) or GAPDH (1:1000). The membranes were washed and incubated with fluorescent secondary antibodies (Alexa Fluor 680 or IRDye 800, Rockland Immunochemicals, Gilbertsville, PA, US) for 1 hr at room temperature at 1:5000, blots were analyzed with the Odyssey infrared imaging system and Odyssey software.

### Co-culture macrophages with tumor cells in three-dimensional peptide gel

Macrophages were isolated from tibias and femurs of 8-week-old WT and CX3CR1^−/−^ mice as previously described in our lab [17]. Three-dimensional peptide gel co-culture was also as described [17]. Macrophages and SL4 cells were mixed at a ratio of 1:2 in peptide gel at a final peptide gel concentration of 3%. Peptide gel co-culture was maintained in DMEM containing 10% FCS at 37°C in a humidified atmosphere containing 5% CO_2_. At 48 hrs after co-culture, peptide gel cultures were fixed in 4% paraformaldehyde in PBS for 15 mins at room temperature and washed for 20 mins in PBS. Frozen sections, 7 μm, of peptide gel cultures were treated with 0.15 M glycine in PBS at 4°C overnight to reduce autofluorescence.

### Matrigel plugs

700 μl of Matrigel (Becton Dickinson) was injected subcutaneously in the ventral area in WT or CX3CR1^−/−^ mice. Matrigel plugs contained 5 × 10^5^ WT or CX3CR1^−/−^ macrophages. Plugs containing PBS were used as a negative control, whereas plug containing 100 ng/ml bFGF (R&D Systems), a known angiogenic factor, was used as a positive control. Each experimental condition was analyzed in triplicate. 7 days after plug implantation, plugs were collected, immediately fixed in 10% buffered formalin. Identification of angiogenesis was performed by immunohistochemistry with the primary antibody against CD31.

### Statistics

Data analysis involved use of GraphPad software (GraphPad Prism version 5.00 for Windows, GraphPad Software). Results are expressed as mean ± SEM. Differences were analyzed by *t* test or ANOVA, and results were considered significant at a *P*<0.05.

## Abbreviations

TAMs: Tumor-associated macrophages; TGF: Transforming growth factor; VEGF: Vascular endothelial growth factor; CXCR: Chemokine (C-X-C motif) receptor; CCR: C-C chemokine receptor; VEGFR: Vascular endothelial growth factor receptor.

## Competing interests

The authors declare that they have no competing interest.

## Authors’ contributions

JZ and MY equally participated in the design and execution of the overall study. JS carried out the pathologic analysis. YM performed *in vitro* experiments. JH and JD were involved in the conception and design of the study as well as in drafting and revising the manuscript. All authors read and approved the final manuscript.
